# Supplementary material for: SOX4 facilitates PGR protein stability and FOXO1 expression conducive for human endometrial decidualization
Source: eLife. 2022 Mar 4;11:e72073. doi: 10.7554/eLife.72073 (PMC8923662; doi:10.7554/eLife.72073)
Supplement: Supplementary file 1. [file elife-72073-supp1.docx]

**Supplementary file 1A. Detailed information of participants for tissue collection in this study.**

|  | Control(n=12) | EMS-RIF(n=12) | P |
| --- | --- | --- | --- |
| Age (years) | 32.62±3.50 | 32.65±3.43 | >0.05 |
| BMI (kg/m2) | 22.01±2.62 | 21.99±3.21 | >0.05 |
| Basal FSH (IU/l) | 7.00±2.23 | 7.05±3.50 | >0.05 |
| Duration of infertility (years) | 0 | 5.61±3.35 | >0.05 |
| No. of ET failures | 0 | 5.50±2.00 | >0.05 |
| Endometrial thickness (mm) | 11.29±3.01 | 10.89±2.65 | >0.05 |

**Supplementary file 1B. Detailed information of participants for primary endometrial stromal cells in this study.**

|  | Control1 | Control2 | Control3 | EMS1 | EMS2 | EMS3 |
| --- | --- | --- | --- | --- | --- | --- |
| Age (years) | 31 | 32 | 33 | 32 | 32 | 34 |
| BMI (kg/m2) | 21 | 22 | 22 | 21 | 22 | 22 |
| Basal-FSH (IU/l) | 6 | 7 | 6.5 | 6.2 | 7.2 | 6.6 |
| Duration of infertility (years) | 0 | 0 | 0 | 2 | 3 | 3 |
| Menstrual cycle | 28 | 30 | 30 | 30 | 32 | 28 |
| Day of menstruation (endometrial sampling time) | 8 | 9 | 7 | 7 | 9 | 8 |
| Endometriosis grade | 0 | 0 | 0 | VI | VI | VI |
