# Supplementary material for: SOX4 facilitates PGR protein stability and FOXO1 expression conducive for human endometrial decidualization
Source: eLife. 2022 Mar 4;11:e72073. doi: 10.7554/eLife.72073 (PMC8923662; doi:10.7554/eLife.72073)
Supplement: Supplementary file 2. [file elife-72073-supp2.docx]

**Supplementary file 2. The sequence of oligonucleotide.**

|  | **Forward primer (5’-3’)** | **Reverse primer (5’-3’)** |
| --- | --- | --- |
| **CRISPR/Cas9 sgRNA** |  |  |
| SOX4: sgRNA | CACCCGAGAACACGGAAGCGCTGC | AAACGCAGCGCTTCCGTGTTCTCG |
| **siRNA sequence** |  |  |
| siRNA sequence-SOX4 | CGACAAGATCCCTTTCATT | / |
| siRNA sequence-FOXO1 | TTCGGAATGACCTCATGGA | / |
| siRNA sequence-HERC4 | CCUUUGGGCAGCUAGGUUU | / |
| siRNA sequence-PGR | CCAGCATGTCGCCTTAGAA | / |
| **ChIP-qPCR primer** |  |  |
| SOX4 | AGGCTGGTCTCGAACTCCT | TCAAATGCCAGACACACTCC |
| FOXO1 | CCGACCTCCCATAACAGAGAA | GCCCTACACGTCATTCTTCTAG |
| PRL | GGTTTCTGATACACTGGCCC | ATGGAAGTCCCGACCAGAC |
| FOSL2 | TGGCAACGTGCGCCAAT | CTGCCCGAGATGAGTCACTA |
| STAT3 | CGCAGAGGTGTTATGTTGCG | TAGGTGACCAAGTAGCCGGA |
| **Luciferase reporter** |  |  |
| SOX4 promoter-vector | AGGCTGGTCTCGAACTCCT | CTAATTCAAATGCCAGACAC |
| **QPCR primer** |  |  |
| GAPDH | ACGGATTTGGTCGTATTGGG | CGCTCCTGGAAGATGGTGAT |
| IGFBP1 | AGAGTCGTAGAGAGTTTAGC | ACACTGTCTGCTGTGATAA |
| PRL | CTACATCCATAACCTCTCCTCAG | GGGCTTGCTCCTTGTCTTC |
| FOXO1 | GGCAGCCAGGCATCTCAT | TGGGTCAGGCGGTTCATAC |
| PGR | TGTATTTGTGCGTGTGGGTG | TACAGCCCATTCCCAGGAAG |
| HERC4 | AAACCAGAGCAGGTTGTTGC | CAGAATCGAGACCCCAAGCA |
| RIF213 | GGCATGTCTTTCTCCCGCAA | CAATGCCTGTGACCCTGGAT |
| UBR3 | ACCACAGGTTTCCGAGGCAG | CGGCTGCACAGGTATCCCAA |
| SOX4 | CCCAGCAAGAAGGCGAGTTA | CATCGGCCAAATTCGTCACC |
