# Supplementary material for: SOX4 facilitates PGR protein stability and FOXO1 expression conducive for human endometrial decidualization
Source: eLife. 2022 Mar 4;11:e72073. doi: 10.7554/eLife.72073 (PMC8923662; doi:10.7554/eLife.72073)
Supplement: Supplementary file 3. [file elife-72073-supp3.docx]

**Supplementary file 3. Plasmids used in this study.**

| 1 | pLVML-FLAG-SOX4-IRES-puro |
| --- | --- |
| 2 | pLVX-HA-IRES-ZSgrenn-SOX4 |
| 3 | pENTER-FLAG-HERC4 |
| 4 | pLVX-FLAG-HERC4-IRES-Zsgreen |
| 5 | HA-pCMV-PGR |
| 6 | pLVX-MYC-PGR-IRES-Zsgreen |
| 7 | MYC- pCMV -PGR-F1 |
| 8 | MYC- pCMV -PGR-F2 |
| 9 | MYC- pCMV -PGR-F3 |
| 10 | MYC- pCMV -PGR-F4 |
| 11 | HA-pCMV-PGR1（565K-R） |
| 12 | HA-pCMV-PGR1（565K-R） |
| 13 | HA-pCMV-PGR1（588K-R） |
| 14 | HA-pCMV-PGR1（592K-R） |
| 15 | HA-pCMV-PGR1（613K-R） |
| 16 | HA-pCMV-PGR1（617K-R） |
| 17 | HA-pCMV-PGR1（626K-R） |
| 18 | HA-pCMV-PGR1（638K-R） |
| 19 | HA-pCMV-PGR1（640-641K-R） |
| 20 | pRK5-HA-Ubiquitin-WT |
| 21 | pcDNA-MYC-Ubiquitin-WT |
| 22 | SOX4 promoter-pGL3 |
